# Roles of the Chemokine System in Development of Obesity, Insulin Resistance, and Cardiovascular Disease

**DOI:** 10.1155/2014/181450

**Published:** 2014-03-16

**Authors:** Longbiao Yao, Oana Herlea-Pana, Janet Heuser-Baker, Yitong Chen, Jana Barlic-Dicen

**Affiliations:** Cardiovascular Biology Program, Oklahoma Medical Research Foundation, 825 NE 13th Street, Oklahoma City, OK 73104, USA

## Abstract

The escalating epidemic of obesity has increased the incidence of obesity-induced complications to historically high levels. Adipose tissue is a dynamic energy depot, which stores energy and mobilizes it during nutrient deficiency. Excess nutrient intake resulting in adipose tissue expansion triggers lipid release and aberrant adipokine, cytokine and chemokine production, and signaling that ultimately lead to adipose tissue inflammation, a hallmark of obesity. This low-grade chronic inflammation is thought to link obesity to insulin resistance and the associated comorbidities of metabolic syndrome such as dyslipidemia and hypertension, which increase risk of type 2 diabetes and cardiovascular disease. In this review, we focus on and discuss members of the chemokine system for which there is clear evidence of participation in the development of obesity and obesity-induced pathologies.

## 1. Introduction

**Figure 1:**
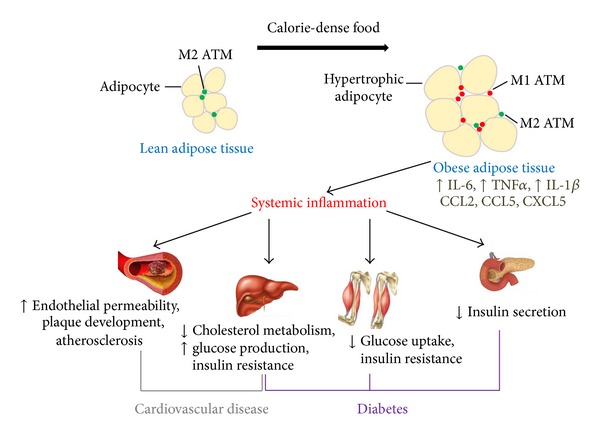
*Obesity contributes to development of diabetes and cardiovascular disease.* Adipose tissue is composed of two main cell types, adipocytes and stromovascular mononuclear cells (i.e., resident leukocytes). Adipose tissue macrophages (ATMs) are the most frequent leukocyte subtype in fat tissues. Normal adipose tissue is populated with the alternatively activated M2 ATMs. Persistent or frequent consumption of calorie-dense food results in obesity that is associated with increased adiposity which includes adipose tissue hypertrophy and influx of proinflammatory monocytes that mature to classically activated M1 ATMs. Obesity induces production of proinflammatory cytokines (i.e., IL-6, TNF*α*, and IL-1*β*) and several chemokines including CCL2, CCL5, and CXCL5 among others by adipocytes and immune cells trigger adipose tissue inflammation, which when prolonged progresses to systemic inflammation that affects (i) vasculature increasing permeability of endothelium, thereby triggering plaque development and cardiovascular disease; (ii) anabolic actions of insulin and insulin signaling in metabolic tissues including liver and skeletal muscle, causing insulin resistance that manifests as impaired glucose disposal in muscle and altered cholesterol and glucose metabolism in the liver, which in turn triggers hyperinsulinemia, hyperglycemia, and hyperlipidemia that all contribute to type 2 diabetes and cardiovascular disease; and (iii) pancreas, decreasing insulin secretion that leads to hyperglycemia, which is a hallmark of diabetes.

More than 35% of adults and almost 17% of youth in the United States were reported to be obese in 2012 (http://www.cdc.gov/obesity/data). Obesity is an independent risk factor for metabolic disorders including insulin resistance, type 2 diabetes, hypertension, adverse plasma lipid concentrations, and cardiovascular disease (Figure 1) [1]. Given the high prevalence of obesity and its health risks, it is important to understand molecules, signals, and mechanisms leading to impaired and/or aberrant adipose tissue functions that support excessive body weight gain.

Chemokines are small-secreted molecules that mediate cell recruitment by interacting with their cognate chemokine receptors. They were originally described as mediators of leukocyte migration during the immune response. Subsequently, chemokines were discovered to control movement of other cell types in many different contexts. The human chemokine system comprises about 50 chemokines and 20 receptors belonging to the seven-transmembrane G protein-coupled receptor family. Based on the motif patterns involving two amino-terminal cysteine residues, chemokines are classified into the following four subfamilies: CXC, CC, C, and CX3C, where X is any amino acid residue. Chemokines appear to exhibit a high degree of functional redundancy. Some chemokines have a one-to-one specificity (specific receptor), while for others multiple chemokine ligands binding to the same receptor (shared receptor) have been reported. When multiple ligands interact with a single receptor, different responses are produced because ligands have different binding affinities for the receptor [2].

In this review we highlight the chemokine-chemokine receptor axes that facilitate development of obesity by supporting chronic low-grade adipose tissue inflammation and those that are a link and/or a key contributing factor to insulin resistance, type 2 diabetes, and cardiovascular disease.

## 2. The Chemokine System in Development of Obesity

The root cause of obesity is a prolonged imbalance between caloric intake and energy expenditure which results in increased lipid storage and adipose tissue expansion. Adipose tissue is composed of two main cell types, adipocytes and stromovascular mononuclear cells (i.e., resident leukocytes). Adipose tissue occurs in white and brown forms that serve different functions [3].

The main role of brown adipose tissue (BAT) is adaptive thermogenesis, the process of regulated heat production in response to the environmental temperature or diet. There are three subcategories of adaptive thermogenesis. Cold exposure induces shivering thermogenesis in skeletal muscle and nonshivering thermogenesis in brown adipocytes. Overfeeding triggers diet-induced or metabolic thermogenesis, which allows excess energy received in the form of food to dissipate as heat and thereby this form of thermogenesis protects from obesity [4].

White adipose tissue (WAT) provides an important energy depot in the form of stored lipids [3]. In addition to energy storage, WAT serves as an endocrine organ that produces adipokines, signaling molecules secreted by adipocytes, which have multiple effects at both local and systemic levels. Excessive fat uptake results in adipocyte expansion (hypertrophy) and in overproduction and secretion of signals that recruit inflammatory cells into WAT, triggering low-grade chronic inflammation that is mediated by the cells of innate and adaptive immune systems [5, 6].

In normal adipose tissue, the main resident immune cell subtypes are the alternatively activated CD163^+^ and/or CD206^+^ (M2) adipose tissue macrophages (ATMs), regulatory T cells (Treg), and T helper 2 (Th2) cells. These resident leukocytes produce anti-inflammatory interleukin-10 (IL-10) and transforming growth factor *β* (TGF*β*) that maintain adipose tissue homeostasis. Aberrant adipose tissue expansion and hypoxia associated with the excessive adipose tissue growth as well as activation of oxidative stress responses orchestrate the production of proinflammatory cytokines like interleukin-6 (IL-6) and tumor necrosis factor alpha (TNF*α*) as well as chemokines. These signals support influx of proinflammatory monocytes, which in WAT mature to classically activated CD40^+^ and/or CD11c^+^ (M1) ATMs, as well as recruitment of neutrophils, mast cells, natural killer cells, and dendritic cells. In addition to innate immune cells, adaptive response immune cells including CD8 effector memory, T helper 1 (Th1), and B cells are all increased in the adipose tissue during the course of obesity. Thus, overfeeding triggers qualitative and quantitative alterations in the adipose tissue stromovascular fraction that, together with adipocyte hypertrophy, fuel chronic low-grade adipose tissue inflammation that exacerbates obesity [6].

Chemokines including CCL2, CCL5, CCL7, CCL8, CCL11, CCL13, CXCL5, CXCL8, and CXCL10 are upregulated in different depots of adipose tissue. Serum levels of these chemokines are dramatically increased in obese versus lean individuals. Expression of chemokine receptors CCR1, CCR2, CCR3, and CCR5 is elevated on inflammatory cells in omental and subcutaneous adipose tissues of obese patients [7, 8].

There are several mouse models that are used to study development of obesity and obesity-induced comorbidities. The model that most closely resembles human obesity and obesity-linked pathologies including metabolic syndrome is the inbred C57BL/6 strain fed the obesogenic high-fat diet containing 60% fat. While this model is useful for studies addressing development of obesity in humans, there are also differences: (i) mice are typically individually housed and in relatively small cages, which restricts social interaction and physical activity; (ii) the normal housing temperature is 20–23°C, which is several degrees lower than their thermoneutral temperature (29–32°C), and animals consequently expend energy to keep warm; (iii) food is readily available at all times, which is not always true for humans; and (iv) eating habits and psychological factors that control food intake and overeating in mice and in man may not be the same [9].

Using the mouse model of diet-induced obesity, it was demonstrated that targeted deletion of the chemokine* Ccl2* [10, 11] or its receptor* Ccr2* [12] decreased inflammatory ATM content and adipose tissue inflammation. Conversely, adipose tissue-specific overexpression of CCL2 increased inflammatory ATM content in adipose tissues [13]. Thus, the CCL2-CCR2 axis was suggested to be the main promoter of inflammatory cell recruitment into fat tissue in obese mice. However, recent studies have produced opposite results. Genetic inactivation of CCL2 was demonstrated to not interfere with obesity-associated monocyte influx into WAT [14]. Furthermore,* Ccr2* ^−/−^ mice fed an obesogenic high-fat diet had fewer macrophages in WAT relative to control wild-type mice; however, lack of CCR2 did not decrease ATM content to the levels detected in lean mice [14, 15]. This indicates that proinflammatory monocyte influx into hypertrophic adipose tissue is, in addition to the CCL2-CCR2 axis, also regulated by other chemokine-chemokine receptor interactions.

Indeed, the chemokine receptor* Cxcr2* ^−/−^ bone marrow chimeras show decreased obesity-induced adipose tissue inflammation [16]. Furthermore, the chemokine receptor CCR5-mediated signaling in the adipose tissue is thought to maintain obesity-induced inflammation. As in obese individuals [17], expression of CCR5 and its ligands is greatly increased in the WAT of high-fat diet-fed obese mice. Furthermore, high-fat diet feeding causes a robust increase in CCR5^+^ ATMs in hypertrophic WAT. Moreover, lack of CCR5 expression in myeloid leukocytes alone is associated with a marked reduction in proinflammatory monocyte infiltration into WAT and protects mice from the obesity-induced adipose tissue inflammation [18]. These data suggest that CCR5^+^ ATMs contribute to development of obesity and obesity-induced adipose tissue inflammation. However, a study by Kennedy et al. shows that CCR5 has a minor role in regulating M1 ATMs infiltration but increases influx of CD4^+^ T into hypertrophic adipose tissue [19], indicating that targeting CCR5 only may not be the best approach for treating obesity-induced adipose tissue inflammation.

## 3. The Chemokine System in Insulin Resistance

Insulin, a hormone produced by *β*-cells in the pancreas, is central in regulation of carbohydrate and fat metabolism. Insulin regulates uptake of blood glucose by metabolic tissues including liver, skeletal muscles, and fat tissues. Obesity compromises metabolic homeostasis by altering glucose absorption and insulin sensitivity in these tissues.

The obesity-induced macrophage phenotype switch in expanding adipose tissue involves a decrease in anti-inflammatory M2 ATM content paralleled with an increase in proinflammatory M1 ATM content. Subsequent recruitment of other innate and adaptive immune cells together with the macrophage phenotype switch triggers systemic inflammation, which decreases insulin sensitivity in adipose as well as in other insulin-sensitive metabolic tissues. Prolonged and/or long-term nutrient excess exacerbates adipose tissue inflammation which interferes with anabolic actions of insulin and insulin signaling in metabolic tissues, causing insulin resistance that manifests as impaired glucose disposal in muscle and enhanced triglyceride lipolysis in adipose tissue. Hyperinsulinemia, hyperglycemia, and hyperlipidemia, which often are a result of insulin resistance, all contribute to metabolic syndromes including development of type 2 diabetes and cardiovascular disease, both of which are global health problems (Figure 1) [20].

Because insulin resistance has a pivotal role in the pathogenesis of type 2 diabetes, efforts are being made to elucidate factors responsible for obesity-induced insulin resistance. Several lines of evidence suggest that the chemokine system links obesity to insulin resistance by regulating macrophage functional responses as well as by controlling proinflammatory monocyte influx and, with that, M1 ATM content in adipose tissues [21].

Genetic inactivation of CCL2-CCR2 signaling [11, 12, 22] or inhibition of this axis [23, 24] by pharmacological approaches was shown to improve insulin sensitivity in obese mice, whereas overexpression of CCL2 impaired glucose metabolism in this mouse model [13]. However, recent data suggest that lack of CCL2 does not improve metabolic function [15]. Furthermore, CCR2 deficiency was not shown to eliminate insulin resistance. Together this shows that glucose metabolism is regulated by CCL2-CCR2 independent signals as well [25].

Deficiency in CCR5 was also shown to improve obesity-induced insulin resistance in mice.* Ccr5* ^−/−^ mice and* Ccr5* ^−/−^ bone marrow chimeras fed the obesogenic diet both showed resistance to obesity-induced insulin resistance and type 2 diabetes [18]. Moreover, myeloid leukocyte CCR5 deficiency alleviated high-fat diet-induced insulin resistance and hepatic steatosis [18]. In contrast to this study, Kennedy et al. recently reported that obese* Ccr5* ^−*/*−^ mice develop systemic glucose intolerance [19]. Thus, inhibition of CCR5 signaling can be considered as a novel intervention to treat insulin resistance and type 2 diabetes only after roles of CCR5 in human adipose tissue are fully identified.

Studies have shown that CXC motif chemokine ligand 5 (CXCL5) and its cognate chemokine receptor CXCR2 also support development of insulin resistance. CXCL5 serum levels are markedly increased in obese compared to lean subjects [26]. Treatment of obese, insulin-resistant mice with either neutralizing anti-CXCL5 antibodies or CXCR2 antagonist decreased fasting glucose levels and improved insulin sensitivity.* Cxcr2* ^−/−^ mice were shown to be protected from diet-induced insulin resistance and diabetes [16, 26]. Thus, this finding suggests that inhibition and/or neutralization of CXCL5 may be considered as a therapeutic tool for treatment of metabolic syndrome.

The expression of the CXC motif chemokine ligand 14 (CXCL14) was shown to be elevated in the high-fat diet-fed WAT of obese mice.* Cxcl14 *inactivation improved insulin sensitivity in high-fat diet-fed female but not male mice. In addition,* Cxcl14* ^−/−^ mice were also protected from diet-induced hyperglycemia, hyperinsulinemia, and hypoadiponectinemia. Interestingly, overexpression of CXCL14 in skeletal muscle restored obesity-induced insulin resistance in* Cxcl14* ^−/−^ mice [27], suggesting that CXCL14 is important during glucose uptake in skeletal muscle and by doing so this chemokine regulates glucose metabolism.

Although the chemokine system is thought to participate in the development of insulin resistance by sustaining adipose tissue inflammation, the CX3C chemokine ligand 1 (CX3CL1) and its cognate chemokine receptor CX3CR1 were demonstrated to regulate pancreatic *β*-cell insulin secretory pathway in which CX3CL1 stimulates CX3CR1 to increase insulin secretion. High-fat diet failed to trigger obesity in* Cx3cr1* ^−/−^ mice and the CX3CL1-CX3CR1 axis did not seem to promote inflammatory macrophage accumulation in adipose tissue or liver, thereby preventing inflammation-induced insulin resistance. However,* Cx3cr1* ^−/−^ mice fed either regular chow (10% fat) or a high-fat diet developed glucose intolerance while exhibiting normal insulin sensitivity. This suggests that deficient insulin secretion causes hyperglycemia in* Cx3cr1* ^−/−^ mice. Furthermore, islets isolated from* Cx3cr1* ^−/−^ mice produced less insulin in response to various stimuli.* In vivo* administration of soluble CX3CL1 increased plasma insulin levels and improved glucose tolerance [28]. Thus, this finding suggests that it may be possible to both improve glucose metabolism and prevent type 2 diabetes by facilitating CX3CR1 signaling.

## 4. The Chemokine System in Cardiovascular Disease

### 4.1. Chemokines and Chemokine Receptors Promoting Plaque Development

The link between obesity and atherosclerotic cardiovascular disease extends beyond the overlapping incidence. These two conditions share similar pathophysiological pathways. Obesity and atherosclerosis used to be described as simple lipid-storage diseases involving triglycerides in adipose tissue and low-density lipoprotein (LDL), also known as “bad cholesterol,” in atheroma. However, obesity and atherosclerosis are now viewed as chronic inflammatory processes supported by activation of innate and adaptive immunity [29–31].

Deposition and modifications of LDL in the subendothelium and the consequent proinflammatory reactions of cellular elements in the arterial wall promote influx of inflammatory leukocytes into the vascular subendothelium, which supports the development of multifocal atherosclerotic plaques. Most plaques are asymptomatic and some become obstructive but only a few become thrombosis-prone and cause complications including acute myocardial infarction and stroke [32, 33].

Statins are widely used to treat atherosclerosis. Large randomized clinical trials have documented their benefits in patients at risk for or presenting established atherosclerotic cardiovascular disease. Statins lower plasma cholesterol by reversibly inhibiting 3-hydroxy-3-methylglutaryl coenzyme A (HMG-CoA) reductase, the rate-limiting enzyme in cholesterol biosynthesis in the liver. They have also been shown in both experimental and clinical studies to have potent anti-inflammatory, vasodilatory, and antiplatelet effects that are independent of their lipid-lowering effects. These non-lipid-lowering effects depend on HMG-CoA reductase inhibition in tissues other than the liver. The effects include improvement of endothelial function, decreased vascular smooth muscle cell proliferation, attenuated vascular inflammation, increased plaque stability, and prevention of thrombus formation. Despite numerous beneficial effects, recent studies investigating effects of long-term intensive statin therapy on atherosclerosis burden have demonstrated that statins do not eliminate plaque burden completely, leaving patients prone to adverse cardiovascular events [34, 35]. Thus, efforts are being made to develop new interventions that will eliminate this risk. For this purpose, suitable preclinical models that allow identification of mechanisms, molecules, and signals supporting plaque development have been generated.

Atherosclerosis-prone apolipoprotein E-deficient (*ApoE* ^−/−^) and low-density lipoprotein receptor-deficient (*Ldlr* ^−/−^) mice have been developed and extensively used in studies of atherosclerotic plaque development and used to evaluate novel drug targets. The use of these models also showed that there are differences between mouse and human atherogenesis. Lesions in mice are not prone to spontaneous plaque disruption with thrombosis that complicates human disease. Mouse studies mostly focus on aortic plaques, whereas the most important clinical consequences of atherosclerosis in humans arise from lesions in the coronary, carotid, and/or cerebral arteries. The proximal left anterior descending coronary artery in humans which is a frequent site of lesion formation contains a considerable population of intimal smooth muscle cells, which is a major difference from mouse arteries. Despite these differences, consumption of the Western diet, dense in fat and carbohydrates, triggers hypercholesterolemia and vascular inflammation that drive plaque development in mice and humans [36, 37].

Inflammatory cells involved in atherogenesis are highly heterogeneous. In mice, a proinflammatory subset of monocytes induced by hypercholesterolemia preferentially furnishes the precursors of lesional macrophages and foam cells, but the fates and functions of this monocyte subset and its human equivalent remain under intense investigation. Plaque macrophages also have proinflammatory functions, characteristic of M1 ATMs in inflamed obese adipose tissue. They produce high levels of proinflammatory cytokines including interleukin-1*β* (IL-1*β*) and TNF*α*. Some mononuclear phagocytes in plaques perform functions of the antigen-presenting dendritic cells. Other leukocyte classes including T and B lymphocytes and mast cells accumulate mostly in advanced atheroma, but these cells are less abundant than phagocytes. Lesional Th1 cells are proatherogenic and Tregs are atheroprotective. The roles of Th2 and Th17 still remain controversial. The B1 subset of B cells is thought to be atheroprotective, and the B2 subset of B cells seems to be proatherogenic [38].

Chemokine-mediated recruitment and accumulation of leukocytes in the arterial wall is an important mechanism supporting atherosclerosis progression. Several chemokines including CCL2, CCL5, and CX3CL1 as well as their cognate chemokine receptors CCR2, CCR5, and CX3CR1 are expressed in mouse and human atherosclerotic lesions [39–44]. Proatherogenic* ApoE* ^−/−^ and* Ldlr* ^−/−^ strains fed the Western diet (21% fat, 0.3% cholesterol) that are deficient in CCL2 [45] or CCR2 [46, 47] show reduced atherosclerosis burden compared to wild-type controls. Single nucleotide polymorphisms (SNPs) in the promoter of CCL2, CCL2-2518G [48], and in the open reading frame of CCR2, CCR2-V64I [49], have been associated with increased risk of myocardial infarction in humans. Furthermore, antagonism or deficiency in CCR5 or CCL5 or CX3CL1 or CX3CR1 also reduces atherosclerosis in mice [50–54]. In humans, the gain-of-function polymorphism CCL5-G403A affects basal CCL5 expression levels and is associated with a higher risk of coronary artery disease (CAD) [55, 56], whereas the loss-of-function polymorphism CCR5Δ32 seems to be associated with a lower risk of myocardial infarction [56, 57]. The analysis of Framingham Heart Study Offspring Cohort indicated that two SNPs of CX3CR1, V249I and T280 M, are associated with a markedly reduced risk of CAD [58–60]. However, two other studies showed that these polymorphisms were not associated with peripheral arterial disease but were associated with increased risk of restenosis after coronary stenting [61, 62].

The main leukocyte subset initiating and driving plaque progression is monocytes. Expression of CCR2, CCR5, and/or CX3CR1 supports differential monocyte subset recruitment and functions in the plaque. Ly6C^hi^CCR2^+^CX3CR1^low^ monocytes (or CD14^hi^CD16^−^ in humans) more efficiently infiltrate sites of inflammation (inflammatory monocytes), while Ly6C^low^CCR2^−^CX3CR1^hi^ monocytes (or CD14^+^CD16^+^ in humans) have a major surveillance function in homeostasis (resident monocytes). Ly6C^hi^ monocytes are dramatically increased in hypercholesterolemic mice. This monocyte subset uses in addition to CCR2 also CCR5 and CX3CR1 to home to plaques. Interestingly, Ly6C^low^ monocytes can also enter plaques, although they do so less frequently than Ly6C^hi^ monocytes and seem to require CCR5 rather than CX3CR1. Although both Ly6C^hi^ and Ly6C^low^ monocyte subsets can differentiate into CD11c^+^ dendritic cells, Ly6C^low^ monocytes are more prone to becoming CD11c^+^ cells within lesions, indicating functional differences between these two monocyte populations [63, 64]. In addition, CX3CL1 is a unique chemokine that exists in both membrane-tethered and soluble forms, thus mediating not only cell recruitment but also adhesion [65]. Evidence has indeed shown that CX3CL1-CX3CR1 interaction mediates firm adhesion of monocytes to smooth muscle cells, which is thought to be at least one mechanism supporting plaque development [66, 67].

Roles of several other chemokine-chemokine receptor axes including CCL20-CCR6, CCL17-CCR4, CXCL1-CXCR2, and CXCL10-CXCR3 in atherosclerosis progression were identified in the mouse models. However, it remains unclear whether these members of the chemokine system, although expressed in atheroma and/or increased in the circulation of hypercholesterolemic and/or CAD patients, contribute to plaque development in humans.

In mice, CCL20 and CCR6 are expressed in healthy vessels and in atherosclerotic plaques [68, 69]. Deletion of* Ccr6* decreased atherosclerosis burden in the* ApoE* ^−/−^ mouse, which was accompanied by lower macrophage content in plaques, suggesting that CCR6 is proatherogenic. Moreover, reduced atherosclerosis in* Ccr6* ^−/−^ bone marrow chimeras fed atherosclerosis-inducing diet indicates that CCR6^+^ hematopoietic cells contribute to lesion development [70]. This finding is consistent with observations in* Ccr6* ^−/−^ *Ldlr* ^−/−^ mice, which showed that Western diet triggered atherosclerosis in this strain; however, plaque burden in* Ccr6* ^−/−^ *Ldlr* ^−/−^ mice was lower than in the* Ldlr* ^−/−^ controls. CCR6 was also shown to promote monocyte adhesion to inflamed endothelium* in vitro* and leukocyte adhesion to carotid arteries* in vivo*. More importantly, CCR6 selectively recruited monocytes but not T cells in an acute inflammatory air pouch model [71]. Thus, because CCR6 controls adhesion and recruitment of monocytes/macrophages to the inflamed vessel, this chemokine receptor may function on multiple levels during plaque development.

CCL17, which is produced by dendritic cells (DCs) and acts through its cognate chemokine receptor CCR4 [72], is upregulated in advanced mouse plaques [73]. Deficiency in* Ccl17* in* ApoE* ^−/−^ mice resulted in reduced atherosclerosis that was supported by immunoregulatory functions of Treg cells. Coculture of CD4^+^ T cells with* Ccl17* ^−/−^ DCs reduced apoptosis and stimulated expansion of Treg lymphocytes, suggesting that dendritic cell CCL17 is a central regulator of Treg homeostasis [74]. This chemokine may support atherosclerosis progression by inhibiting Treg expansion.

CXCR2 and its chemokine ligand CXCL1 are expressed in mouse plaques [75]. Transplantation of* Cxcr2* ^−/−^ bone marrow to* Ldlr* ^−/−^ recipients fed Western diet resulted in smaller lesions, suggesting that hematopoietic cell CXCR2 promotes atherogenesis. Similarly, inactivation of* Cxcl1* decreased atherosclerosis burden in* Ldlr* ^−/−^ mice [76]. Since CXCL1 is mainly produced by macrophages, neutrophils, epithelial, and endothelial cells and because CXCR2 is found on neutrophils, monocytes, and mast cells, the proatherogenic effect of CXCL1-CXCR2 axis is likely due to monocyte arrest on atherosclerotic endothelium and CXCR2-mediated macrophage accumulation in lesions [77]. However, since neutrophils were shown to promote atherosclerosis, the CXCR2-CXCL1 axis may also direct neutrophils to developing plaques thereby promoting atherogenesis.

Genetic inactivation of* Cxcr3* or its ligand* Cxcl10*, that were both detected in mouse lesions in the* ApoE* ^−/−^ strain, resulted in decreased atherosclerosis burden that was accompanied by a decrease in inflammatory CD4^+^ T cell content, an elevated number of Tregs, and increased expression of IL-10 [78]. This finding suggests that the CXCR3-CXCL10 interaction regulates atherosclerosis progression by controlling the recruitment and T cell content in lesions.

Thus, evidence from atherosclerosis-prone mouse models and analysis of human cardiovascular cohorts reinforce the important contribution of multiple chemokines in plaque development.

### 4.2. Atheroprotective Chemokines and Chemokine Receptors

CCR1, a receptor for CCL3 and CCL5, is expressed on human monocytes, macrophages, and T cells and on mouse neutrophils [79]. Reconstitution of irradiated* Ldlr* ^−/−^ mice with* Ccr1* ^−/−^ bone marrow increased atherosclerotic surface in both the thoracic aorta and the aortic root, indicating that CCR1 opposes atherogenesis [80].* Ccr1* ^−/−^ *ApoE* ^−/−^ mice have accelerated atherosclerosis relative to* ApoE* ^−/−^ controls. Increased atherosclerosis burden correlated with a significant increase in CD3^+^ T cell numbers in plaques, whereas lesional macrophage content remained unaffected in these mice [50]. CCR1 was also reported to support CCL5-mediated arrest of monocytes and Th1 cells on activated endothelium [81]. Thus, CCR1 may be protecting from atherosclerosis by preventing excessive T cell and monocyte influx into atherosclerotic vascular wall.

CCR7 promotes homing of T cells and DCs to T cell areas of lymphoid tissues where T cell priming occurs. CCR7 and its ligands CCL9 and CCL21 contribute to a multitude of adaptive immune functions including thymocyte development, secondary lymphoid organogenesis, high affinity antibody responses, regulatory and memory T cell function, and lymphocyte egress from tissues [82]. CCR7 and CCL19/CCL21 are expressed in mouse and human atherosclerotic plaques [83], suggesting that CCR7-CCL19/CCL21 interaction is important in atherogenesis. CCR7 deficiency in* ApoE* ^−/−^ mice exacerbated atherosclerosis, which correlated with a selective increase in circulating T cells and their accumulation in atherosclerotic plaques.* Ccr7* ^−/−^ *ApoE* ^−/−^ mice had increased Th1 and Th17 responses but reduced Th2 and Treg responses [84]. Thus, evidence shows that CCR7 in atherogenic conditions opposes plaque development.

CXCR4 is constitutively expressed on a variety of different cell types. During embryogenesis CXCR4 and its chemokine ligand CXCL12 regulate development of hematopoietic, cerebellar, and endothelial tissues by controlling tissue progenitor cell migration, homing, and survival [85–87]. In adult life, this axis serves as the key factor for physiologic stem cell and immune cell trafficking [88–90]. Plasma CXCL12 levels in CAD patients are lower than in healthy controls [91]. Aged* ApoE* ^−/−^ mice with advanced atherosclerosis have lower levels of CXCL12 in serum and bone marrow than their age matched controls [92], indicating that CXCR4-CXCL12 axis may counter plaque development. Treatment of* ApoE* ^−/−^ mice with the CXCR4 antagonist AMD3465, reconstitution of* ApoE* ^−/−^ mice with* Cxcr4* ^−/−^ bone marrow, or repopulation of* Ldlr* ^−/−^ mice with bone marrow overexpressing “degrakine,” which traps CXCR4 in the endoplasmic reticulum, all increased plaque burden. This increase in atherosclerosis burden was associated with neutrophilia and with increased neutrophil content in plaques. More importantly, inhibition of CXCR4 signaling or hematopoietic cell CXCR4 deficiency also coassociated with decreased smooth muscle cell and CD3^+^ T cell content in plaques, suggesting that lack of CXCR4 may increase plaque vulnerability [93]. Thus, the CXCR4-CXCL12 axis plays a protective role in regulating neutrophil infiltration into the atherosclerotic plaques. Since CXCR4 is conserved across species, understanding how this chemokine receptor functions in the mouse may explain its role in human atherosclerosis.

### 4.3. Is the Chemokine System Involved in Atherosclerotic Plaque Resolution?

Complete resolution of atherosclerosis is a desired clinical goal. However, mechanisms supporting regression of plaques remain obscure. This is due in part to a limited number of preclinical atherosclerosis-prone models in which hypercholesterolemia and vascular inflammation, which are the main drivers of plaque development, can be effectively reversed.

As one approach to this problem, the adenovirus-mediated restoration of apolipoprotein E (ApoE) expression in* ApoE* ^−/−^ mice was shown to reverse hypercholesterolemia and reduce foamy macrophage burden in lesions. The main mechanism supporting regression of plaques in this model is reduced monocyte entry into lesions combined with a stable rate of macrophage apoptosis [94]. If the gene transfer strategy is used to achieve reversal of hypercholesterolemia, regression of atherosclerosis occurs only under sustained, high level ApoE expression, which is yet to be achieved in the clinical setting.

Another model to study atherosclerosis regression involves transplantation of plaque-containing portions of aorta from hypercholesterolemic* ApoE* ^−/−^ mice into normolipidemic C57BL/6 recipients. In this model, normal plasma cholesterol levels in recipients triggered plaque regression that coincided with rapid emigration of inflammatory cells from plaques [95]. The experimental design in this investigation rapidly eliminated hypercholesterolemia and disrupted lymphatic and arterial systems. This approach to normalize plasma lipids does not mimic statin-induced changes in patients and because it is acute and invasive, it may trigger responses that are stress-induced rather than triggered by normalization of plasma cholesterol thereby limiting the translational potential of this model.

Moderate atherosclerosis regression was observed in the mouse model in which hypercholesterolemia was reduced by switching atherogenic mice fed the Western diet to regular chow diet [96] as well as in hypercholesterolemic mice treated with liver-X-receptor agonist [97] or high-density lipoprotein [98].

Atherosclerotic plaque regression was also observed in the Reversa model (*Ldlr* ^−/−^ *ApoB* ^*100/100*^ *Mttp* ^*fl/fl*^ *Mx1*-*Cre*, C57BL/6 background) [99, 100]. Reversa mice lack low- density lipoprotein receptor and express atherogenic apolipoprotein B 100. The Western diet-induced hyperlipidemia causes aortic atherosclerosis reminiscent of that in* ApoE* ^−/−^ and* Ldlr* ^−/−^ strains. However, hypercholesterolemia is reversed upon Cre-dependent inactivation of the microsomal triglyceride transfer protein (*Mttp*), which is required for the transport of neutral lipids to nascent ApoB lipoproteins and the assembly of atherogenic LDL in the liver [101]. The Reversa model is ideal for studying regression of atherosclerosis because genetic inactivation of* Mttp* results in moderate and time-dependent lowering of plasma lipids that mimics statin-induced normalization of plasma lipids in patients.

The chemokine system was found to participate in atheroregression. In the transplant model of atherosclerosis regression, the CCR7-CCL19/CCL21 axis was shown to direct emigration of inflammatory cells from plaques, reducing plaque burden and stabilizing lesions [95, 97]. However, the critical role of CCR7 in atheroregression was not confirmed in the model in which overexpression of ApoE was used to reverse hypercholesterolemia [94]. The controversy between these two models remains unexplained; however, it also remains unclear how above normal plasma ApoE levels affect expression and function of CCR7 and/or CCL19/21. This should be investigated because ApoE, in addition to mediating the uptake of chylomicrons, very low-density lipoprotein (VLDL), and their remnants, also activates signaling cascades suppressing production of proinflammatory cytokines, regulates monocyte/macrophage polarity and was demonstrated to play a pivotal role in maintaining the Th1/Th2 balance [102]. Thus, abnormal levels of ApoE may alter expression and function of signals and receptors engaged in atheroregression, thereby inducing mechanisms of plaque regression that may not be physiologically relevant.

## 5. Conclusion

Given the serious immediate and long-term consequences of the global obesity epidemic, understanding of the mechanisms that support development of obesity and obesity-related comorbidities should be considered a top priority to which is devoted much research interest and effort. Animal experiments have proven indispensable in uncovering mechanisms driving pathophysiology of obesity and atherosclerotic cardiovascular disease. While experimental atherosclerosis and obesity in animals allow the rigorous testing of mechanistic hypotheses, they do not mimic the human condition entirely. Because of this, animal studies require critical evaluation and interpretation and recognition of their limitations. However, animal models, despite being imperfect, have taught us that the pathological mechanisms of obesity recapitulate many features of the inflammatory processes at work in atherosclerosis. Chronic inflammation that drives both obesity and plaque development (Figure 1) is greatly supported by the chemokine system, which makes chemokines and chemokine receptors attractive therapeutic targets for treatment of atherosclerosis or to combat obesity.
